# Next generation meshes for hernia repair: Polypropylene meshes coated with antimicrobial benzalkonium chloride induced proliferative activity of fibroblasts

**DOI:** 10.1016/j.heliyon.2024.e24237

**Published:** 2024-01-06

**Authors:** Ángel Serrano-Aroca, Alba Cano-Vicent, Alberto Tuñón-Molina, Salvador Pous-Serrano

**Affiliations:** a Biomaterials and Bioengineering Lab, Centro de Investigación Traslacional San Alberto Magno, Universidad Católica de Valencia San Vicente Mártir, C/Guillem de Castro 94, 46001, Valencia, Spain; b Surgical Unit of Abdominal Wall, Department of General and Digestive Surgery, La Fe University Hospital, Valencia, Spain

**Keywords:** Hernia repair, Mesh, Polypropylene, BAK, Biocompatibility

## Abstract

Hernia repair is one of the most frequently performed world-wide surgical procedures in which hernia meshes are becoming increasingly used. Polypropylene (PP) mesh implants reduce the risk of recurrence and post-operative pain, although many other risks are associated with it, such as bacterial infection. In this study we developed PP meshes coated with the well-known antimicrobial compound, benzalkonium chloride (BAK) by dip-coating. Several dilutions (40, 20, 30, 10, 7.5, 5, 2.5, 1, 0.5, 0.1 and 0.05 % *v/v*) of commercial BAK solution (BAK diluted in 70 % ethyl alcohol at 0.1 % *w/v*) were used to produce antimicrobial meshes with different amounts of BAK. The dip-coating treatment with low concentrations of BAK (1, 0.5, 0.1 and 0.05 % *v/v* dilutions) was found to have biocompatible results in fibroblast. The use of 0.1 and 0.05 % *v/v* dilutions (PP meshes with up to ∼2 % *w/w* of BAK) showed proliferative activity on fibroblast cells, indicating that these novel antimicrobial meshes show great promise for hernia repair due to their ability to prevent infections while inducing fibroblast proliferation.

## Graphical abstract

**Figure:**
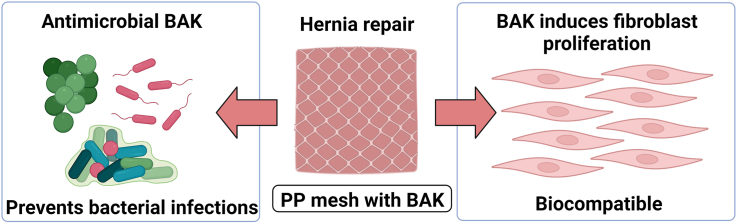

## 1 Introduction

Hernias are among the most prevalent abdominal wall defects that require surgery and include the protrusion of an organ through a weak spot in the cavity of the abdominal wall [1]. Hernias take many different forms according to its position in the body. It can show up in the femoral canal, epigastrium, umbilicus, and inguinal cavities. The most frequently occurring types of hernia are the inguinal (70–75 %), femoral (6–17 %), epigastric (8.6 %), umbilical (3–8.5 %) and incisional (6.2 %), with other kinds such as spigelian [2,3]. They typically cause pain by creating a noticeable protrusion on the skin and sometimes also involve life-threatening complications for patients. More than 20 million hernia repair surgeries are thought to be performed annually around the globe [4]. Due to a number of risk factors, including obesity and prior abdominal surgery, the number of procedures has been rising and is expected to continue to do so [5].

There has been a significant increase in the use of meshes for hernia repair. In this context, surgical meshes are crucial medical equipment that aid in repairing the injured tissue. In order to stabilize the abdominal wall and provide long-term resistance, surgical prostheses for hernia repair are designed to strengthen and replace tissue abnormalities [6]. One of the biomaterials most frequently used in these meshes consists of a variety of natural and synthetic polymers with various structures (reticular, laminar, and hybrid) and characteristics (pore size, filament distribution) [1], polypropylene (PP) being the preferred material to fabricate meshes for these repairs [1]. PP implants reduce the risk of recurrence and post-operative pain [7], although there are many other risks associated with them, like nerve entrapment, mesh erosion, mesh exposure, pain and infection [8]. The high incidence of infection due to these surgical processes has repercussions on factors such as economic aspects, the social burden, hospital re-admissions, re-operations, hernia recurrence, impaired quality of life and plaintiff litigation [9,10]. The infection rate following an open inguinal hernioplasty in a clean field varies between 2.4 % and 4.9 % [11]. These percentages increase if the surgery is clean-contaminated, contaminated, or dirty. It also increases in the case of patients with risk factors such as diabetes, steroid use, obesity, recurrent hernia, etc. In cases of abdominal wall reconstruction due to incisional hernia, surgical site infection rates are close to 33 % [12,13].

By the year 2050, the World Health Organization (WHO) predicts that antibiotic resistance will surpass other significant diseases like cancer as one of the top causes of death [14], so that alternative antimicrobial agents such as quaternary ammonium compounds are being proposed to combat microbial resistance [15].

Meshes loaded with antibiotics have been developed and even though these meshes may have antibacterial activity against bacteria such as *Escherichia coli* and MRSA, they tend to be expensive and do not induce cell proliferation [16], the latter property being important to improve healing after hernia repair surgery [17].

As some recent studies concluded that benzalkonium chloride (BAK), a quaternary ammonium compound, has good antibacterial properties [18,19] we therefore hypothesized that antimicrobial meshes treated with different dissolutions of benzalkonium chloride would be biocompatible (non-cytotoxic at 72 h) and enhance proliferation activity, using fibroblasts as the *in vitro* model.

## 2 Material and methods

### 2.1 Materials

The commercial ultralight macroporous Herniamesh® mesh (REF. H60611, minimum porosity of 410 μm, average of 1800 μm, and maximum of 2270 μm, with a filament diameter of 120 μm and 48 g/m^2^, Chivasso TO, Italia) was used for the study. All the materials were stored and handled according to the manufacturer's instructions.

**Fig. 1:**
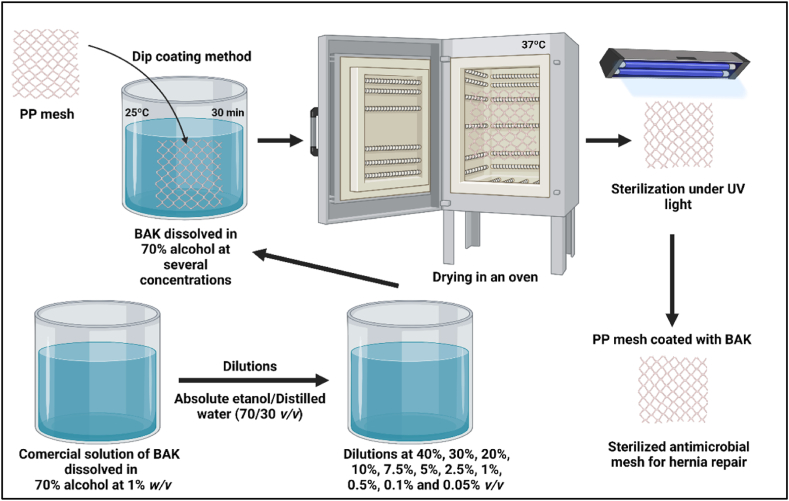
Dip-coating procedure followed to produce antimicrobial PP meshes coated with benzalkonium chloride (BAK).

The mesh was cut into 1 × 1 cm fragments, which were dried at 37 °C for 24 h. A commercial 0.1 % *w/v* BAK solution (Montplet, Barcelona, Spain) was used diluted with absolute ethanol/distilled water (70/30 *v/v*) at 40 %, 30 %, 20 %, 10 %, 7.5 %, 5 %, 2.5 %, 1 %, 0.5 %, 0.1 % and 0.05 % *v/v*. The mesh fragments were treated with the different dilutions by the dip coating method, which is a straightforward, inexpensive, dependable and reproducible technique in which a thin BAK layer is physically adsorbed onto the PP surface [20] for 30 min at 25 °C (see Fig. 1).

These samples, hereafter referred to as 40_BAK, 30_BAK, 20_BAK, 10_BAK, 7.5_BAK, 5_BAK, 2.5_BAK, 1_BAK, 0.5_BAK, 0.1_BAK, 0.05_BAK samples. Six mesh fragments (n = 6) of the concentrations were subjected to the same dip-coating treatment. Six mesh fragments were treated with absolute ethanol/distilled water solution (70/30 % *v/v*) without BAK for 30 min at 25 °C as a control and treated with solvent (S mesh). Six untreated fragments were the untreated control material (U mesh). All the treated and untreated mesh fragments were dried at 37 °C for 24 h and sterilized under UV radiation for 1 h per disk side. Nuclear magnetic resonance was previously performed to characterize the BAK used in the study by means of a BRUKER AVIIIHD 800 MHz (Bruker BioSpin AG, Fälladen, Switzerland) equipped with a 5 mm cryogenic CP-TCI [18].*Toxicological Study*.

The mesh fragments were sterilized for 1 h on each side under an ultraviolet light source. Each biomaterial was evenly distributed in a 6-well plate containing DMEM (Biowest SAS, France) without Fetal Bovine Serum (FBS), following the recommendations of the ISO-10993 standard and the recommended volume ratio of 0.1 g/mL was chosen for irregular porous low-density materials such as textiles.

The extracts were obtained and used right away for the toxicological assay after the mesh fragments were incubated in a humidified 5 % CO2/95 % ambient atmosphere for 72 h at 37 °C. A L929 mouse fibroblast cell line was used in the cytotoxicity tests in which the cells were incubated at 37 °C and 5 % CO2 in a DMEM solution with 10 % FBS, 100 units/mL of penicillin (Lonza, Belgium), and 100 mg/mL of streptomycin (HyClone, GE Healthcare Life Sciences). The MTT assay was used to determine the impact of the mesh fragment treatment on cell viability. The fibroblast cells were seeded at a density of 10^4^ cells per well in a 96-well plate. The media in the wells were replaced with 100 μL of mesh fragment extracts following a 24-h incubation period at 37 °C. The medium was also changed with 100 μL of the same medium used to create the mesh fragment extracts (positive control) and 100 μL of a 1000 μM zinc chloride (97.0 %, Sigma-Aldrich) solution as a negative control as this concentration is highly toxic [21]. 5 mg/mL MTT was used to incubate the cells in each well for 3 h. As a result, 100 μL of dimethyl sulfoxide (Sigma-Aldrich) at room temperature was added to the formazan crystals, after which the absorbance at 550 nm was measured by a microplate reader (Varioskan, Thermo Fisher).

#### 2.1.1 Proliferation study

The mesh fragments were sterilized for 1 h on each side under an ultraviolet light source. No toxic concentrations were studied. The different biomaterials were evenly distributed across the surface of a 6-well plate containing DMEM (Biowest SAS, France) without Fetal Bovine Serum (FBS), following the recommendations of the ISO-10993 standard, by which a volume ratio of 0.1 g/mL was chosen for irregular porous low-density materials.

The extracts were obtained and immediately used for the toxicological assay after the mesh fragments had been incubated in a humidified 5 % CO2/95 % ambient atmosphere for 72 h at 37 °C. A L929 fibroblast cell line was used. The cells were incubated at 37 °C and 5 % CO2 in DMEM containing 0.5 % FBS, 100 units/mL of penicillin (Lonza, Belgium), and 100 mg/mL of streptomycin (HyClone, GE Healthcare Life Sciences). The MTT assay was used to assess the effect of the mesh fragment treatment on cell proliferation. 5 × 10^3^ fibroblast cells were seeded in each well in a 96-well plate. 100 μL of mesh fragment extracts were added to the medium in the wells after 24 h of incubation at 37 °C. 100 μL of the medium used to create the mesh fragment extracts was added as a positive control, and 100 μL of a highly toxic 1000 μM zinc chloride (97.0 %, Sigma-Aldrich) solution was added as a negative control [21]. 100 μL of 15 ng/mL epidermal growth factor (EGF) was added as a proliferation control. Cell incubation was carried out with 5 mg/mL MTT in each well for 3 h after the mesh fragments had been incubated for 72 h at 37 °C in a humidified 5 % CO_2_/95 % air atmosphere. As a result, 100 μL of dimethyl sulfoxide (Sigma-Aldrich) at room temperature was added to the formazan crystals and a microplate reader (Varioskan, Thermo Fisher) was used to measure the absorbance at 550 nm.

### 2.2 Mesh characterization

The amount of BAK physically adsorbed to the PP meshes was determined gravimetrically. Only the meshes that showed biocompatible results at 72 h were characterized. The treated meshes were dried in vacuo to constant weight and weighted after the dip-coating process to determine the amount of BAK adsorbed.

### 2.3 Morphology

The morphology of untreated mesh (U Mesh) and those treated with 0.1 and 0.05 % BAK (0.1_BAK and 0.05_BAK, respectively) was examined on a Leica DM750 optical microscope and Leica ICC50 W images were taken at 4x and 10× magnifications. The images were processed on Leica Application Suite X software (Leica, Madrid, Spain). Macroscopic photographs of the mesh fragments were also obtained by a 24 MP Huawei camera at an opening of f/1.8. Untreated and treated mesh morphology was examined on a field emission scanning electron microscopy (FESEM, Zeiss Ultra 55 Model) with energy-disperse x-ray spectroscopy at an accelerating voltage of 3 kV and magnification of 27, also at a voltage of 10 kV for the elemental analysis. The samples were then sputter-coated with gold.

### 2.4 Statistical analysis

GraphPad Prism 6 software was used for the one-way ANOVA analysis of variance for multiple value comparisons and Tukey's posthoc test (*p > 0.05, ***p > 0.001).

## 3 Results

### 3.1 Toxicological Study

**Fig. 2:**
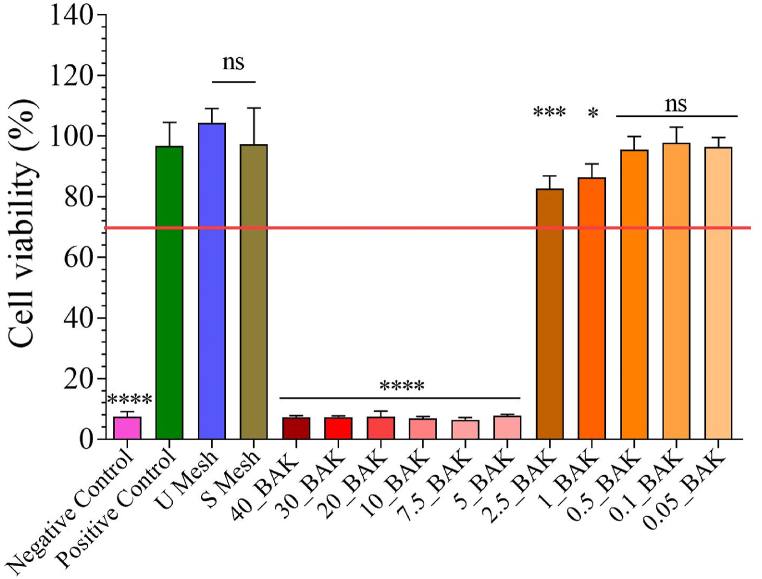
3-[4,5-dimethylthiazol-2-yl]-2,5-diphenyl tetrazolium bromide (MTT) cytotoxicity assay of extracts obtained from control, untreated mesh fragment (U Mesh), mesh fragment treated with 70 % of ethanol (S Mesh), mesh fragment treated by different BAK dissolution (40_BAK, 30_BAK, 20_BAK, 10_BAK, 7.5_BAK, 5_BAK, 2.5_BAK, 1_BAK, 0.5_BAK, 0.1_BAK, 0.05_BAK), positive and negative controls cultured in the presence of fibroblast at 37 °C. The results of the statistical analysis of the positive control are indicated in the graph ***p > 0.001; ns, not significant.

The results of the cytotoxicity tests performed on the extracts in the presence of fibroblast are shown in Fig. 2.

The extracts of 1_BAK, 0.5_BAK, 0.1_BAK, 0.05_BAK samples showed no statistically significant differences in cell viability (%) from the positive control, although the 2.5_BAK had significant differences with cell viability (%), although its cell viability was over 70 %, indicating non-cytotoxicity. The high concentrations of the extracts (40_BAK, 30_BAK, 20_BAK, 10_BAK, 7.5_BAK and 5_BAK) showed statistically significant differences in % of cell viability (lower than 70 %), indicating that the samples were cytotoxic.

#### 3.1.1 Proliferation study

**Fig. 3:**
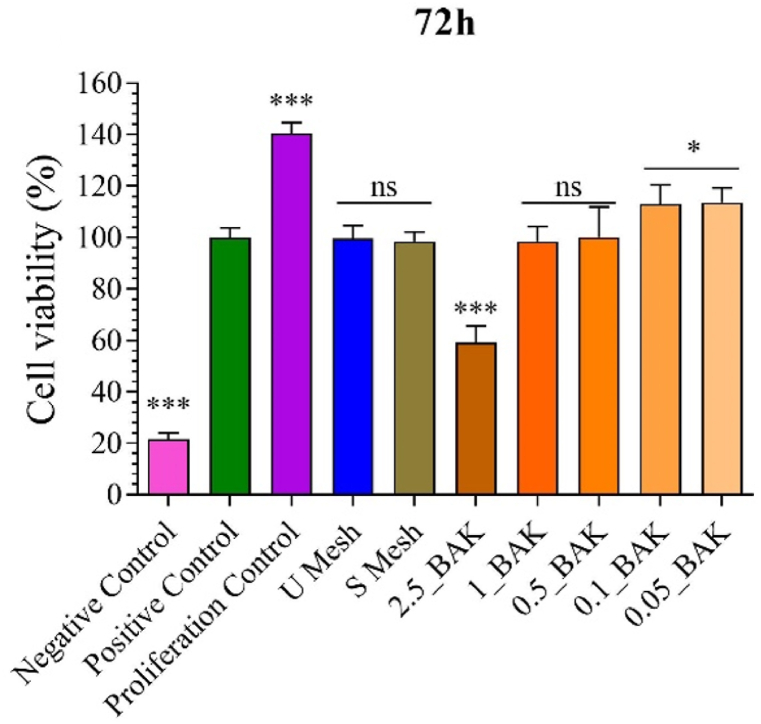
Proliferative activity of mesh fragments with BAK in fibroblast cells stimulated by exposure to non-cytotoxic concentrations for 72 h. The results of the statistical analysis on the positive control are indicated in the graph ***p > 0.001; ns, not significant.

The proliferative activity of mesh fragments with BAK in the fibroblast cell line was studied using non-cytotoxic concentrations (2.5_BAK, 1_BAK, 0.5_BAK, 0.1_BAK and 0.05_BAK) based on the results previously obtained from the cytotoxic assay (Fig. 2) to avoid toxic effects by increasing exposure time to 72 h (Fig. 3).

The results at 72 h showed a statistically significant increase in cell growth in the 0.1_BAK and 0.05_BAK concentrations, while the 2.5_BAK mesh was non-biocompatible after 72 h.

### 3.2 Mesh characterization

The biocompatible amounts of BAK adhered to the PP mesh surface determined gravimetrically are shown in Table 1.

**Table 1:** Biocompatible amounts of BAK adhered to the PP mesh surface determined gravimetrically.

SAMPLE	BAK (% *w/w*)
1_BAK	5.43 ± 1.91
0.5_BAK	3.80 ± 1.61
0.1_BAK	1.98 ± 0.36
0.05_BAK	1.05 ± 0.39

**Fig. 4:**
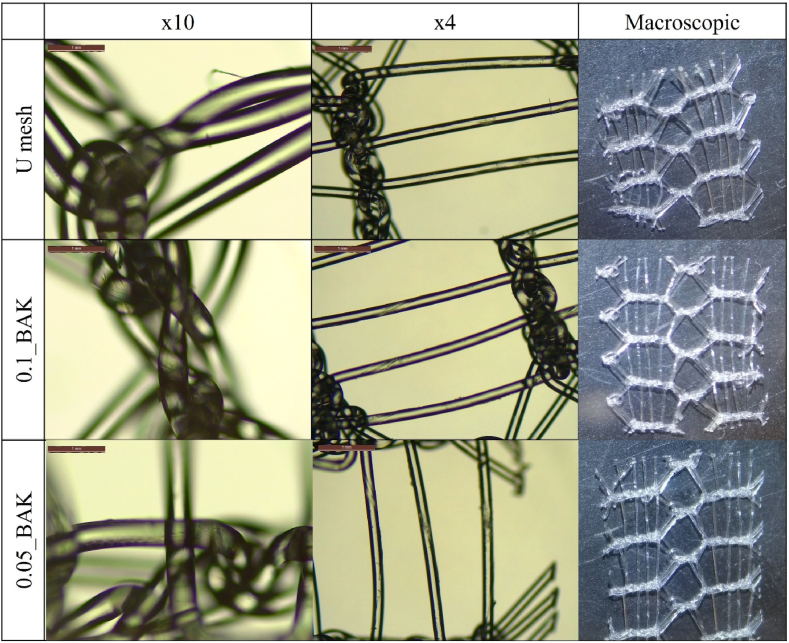
Pictures captured by optical microscope at two different magnifications (x10 and x4), also macrophotographs of the untreated Herniamesh® (U Mesh) and mesh after treatment with 0.1 and 0.05 % BAK (0.1_BAK and 0.05_BAK, respectively) capable of inducing fibroblast proliferation.

The physically adsorbed amounts of BAK on the surface of the mesh filaments are so low that the mesh morphology hardly changes and retains its porous structure (Fig. 4).

**Fig. 5:**
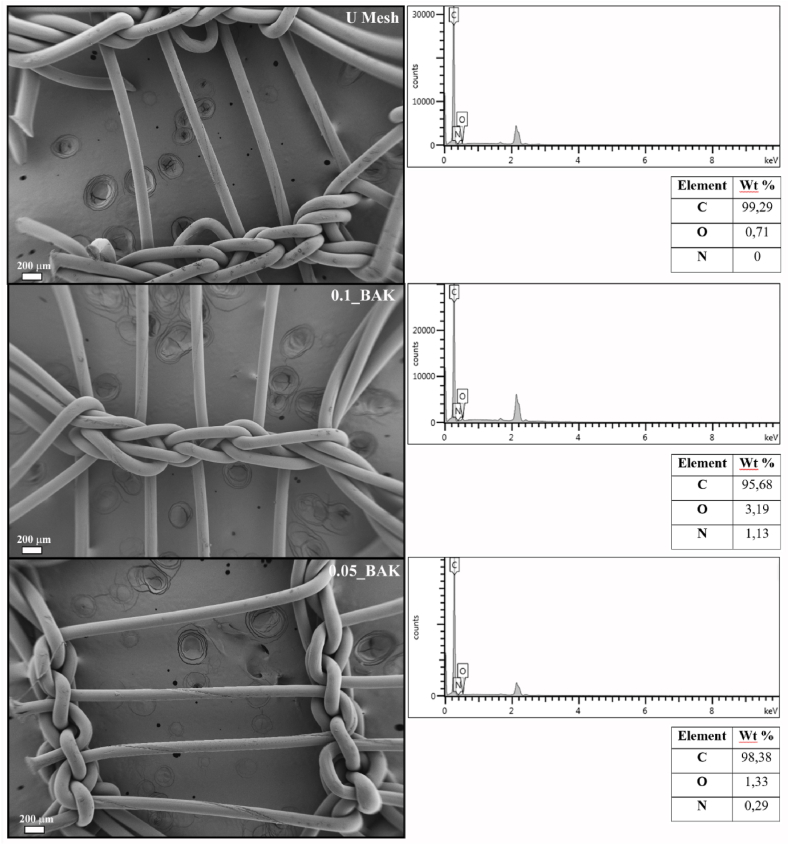
Field emission electron microscopy images at 3 kV and elemental analysis at 10 kV of the untreated Herniamesh® (U Mesh) and mesh after treatment with 0.1 and 0.05 % BAK (0.1_BAK and 0.05_BAK, respectively) capable of inducing fibroblast proliferation. Elemental analysis of the treated and untreated filaments determined by energy-dispersive X-ray spectroscopy. Values expressed as weight percentage.

The pre- and post-treated meshes with 0.1 and 0.05 % BAK were also analyzed by field emission electron scanning microscopy (Fig. 5).

BAK absorption was shown to have no effect on the original mesh morphology (U Mesh). Analysis of the filaments of these three types by EDS showed the presence of nitrogen, i.e. the presence of BAK only on the surface of 0.01_BAK and 0.05_BAK, as expected (Fig. 5).

## 4 Discussion

There are potential complications related to the placement of meshes and patients may, in some instances and in some countries where this is more common, such as the USA or UK, file claims [22,23]. However, without mesh placement, hernia recurrence rates are extremely high [1]. The only controversy lies in whether meshes should be placed in contaminated or dirty fields. The latest publications support their placement and also indicate that synthetic meshes (PP) provide the best results in these cases [24]. The most frequently used mesh worldwide, due to its characteristics, price, infection resistance, tissue integration, etc., is the PP mesh [1]. The only location where PP meshes should not be placed is intraperitoneal (IPOM).

In this study, comercial PP meshes were treated with 0.1 % *w/v* BAK solution diluted with absolute ethanol/distilled water (70/30 *v/v*) at concentrations ranging from 40 to 0.05 % *v/v* (40_BAK, 30_BAK, 20_BAK, 10_BAK, 7.5_BAK, 5_BAK, 2.5_BAK, 1_BAK, 0.5_BAK, 0.1_BAK, 0.05_BAK samples) by the dip coating method [20] (Fig. 1). However, only the extracts of the 2.5_BAK, 1_BAK, 0.5_BAK, 0.1_BAK, 0.05_BAK samples showed non-cytotoxic effect on L929 mouse fibroblast cell line (Fig. 2). BAK is a well-know compound with antimicrobial properties [18,19], although it can be toxic at high concentrations [25,26].

The proliferative activity of the antimicrobial meshes at 72 h was studied in the fibroblast cell line using the non-cytotoxic concentrations (Fig. 3). The results showed that the 0.1_BAK and 0.05_BAK meshes induced fibroblast proliferation. However, the 2.5_BAK mesh showed to be toxic after 72 h.

Table 1 shows that the biocompatible amounts of BAK adhered to the PP mesh surface (determined gravimetrically) range from 1.05 ± 0.39 to 5.43 ± 1.91 % *w/w*.

These physically adsorbed amounts of BAK are so low that the macroscopic, optical microscopy and FESEM images of the meshes show that the polymer structures keep their porous morphology (Fig. 4, Fig. 5).

The presence of BAK in these advanced meshes was demonstrated by FESEM-EDS (Fig. 5). Thus, the EDS results showed the presence of nitrogen atoms on the surface of 0.01_BAK and 0.05_BAK samples (1.13 and 0.29 wt %, respectively). However, the untreated Herniamesh® (U Mesh) showed no nitrogen content as expected. The expected effectiveness on antibacterial properties of these meshes are high because previous dip-coatings of the BAK compound on similar polymer surfaces showed strong antimicrobial activity against multidrug-resistant bacteria [18,19].

It is well-known that increasing biocompatibility of meshes for hernia repair and integration might be the main avenues to achieve better outcomes [27]. The typical progression of the healing process necessitates a flawless coordination of each stage, including hemostasis, inflammation, proliferation, and remodeling, along with the participation of every type of cell [28]. These next generation meshes (0.1_BAK and 0.05_BAK) showed high biocompatibility even after 72 h in fibroblasts. Furthermore, since they were able to increase fibroblast proliferation, these meshes containing antimicrobial benzalkonium chloride will integrate better into the tissues, which is assumed to improve tolerance to acute infection, thereby avoiding the need to remove infected meshes and reducing the possibility of chronic mesh infection (biofilm). This would represent a significant scientific advancement, as it would enhance hernia surgery outcomes, decrease morbidity, lower the likelihood of reoperations, and improve the quality of life for these patients. Costs would also be reduced.

## 5 Conclusions

Polypropylene meshes for hernia repair reduce the risk of recurrence and post-operative pain. However, there are also many risks associated with its use, such as bacterial infections. The present advance provides a new and improved surgical mesh for hernia repair and includes antimicrobial activity and fibroblast proliferating activity. Meshes treated with various concentrations of antimicrobial BAK were studied. The low concentrations of BAK mesh (treated with 1, 0.5, 0.1 and 0.05 % *v/v* dilutions of a commercial BAK solution) proved to be biocompatible with fibroblasts. Treatment with 0.1 and 0.05 % BAK dilutions improved the proliferative activity of these cells. These results are promising due to the ability of these antimicrobial meshes to prevent infections while inducing fibroblast proliferation.

## Author contributions

Á.S-A., A.C.-V. and A.T.-M. performed the experiments; Á.S.-A. got funding, led the work and wrote the draft manuscript; Á.S-A., A.C.-V. and S·P–S edited and proof-read the manuscript. All the authors have read and agreed to the published version of the manuscript.

## Additional information

No additional information is available for this paper.

## Declaration of competing interest

The authors declare the following financial interests/personal relationships which may be considered as potential competing interests:The findings of this study contributed to patent application AX220202EP to the OEPM Office, Madrid, Spain, with Á.S-A. as inventor. The remaining authors declare no competing interests.
